# Biliopancreatic Diversion: The Effectiveness of Duodenal Switch and Its Limitations

**DOI:** 10.1155/2013/974762

**Published:** 2013-11-21

**Authors:** Blaire Anderson, Richdeep S. Gill, Christopher J. de Gara, Shahzeer Karmali, Michel Gagner

**Affiliations:** ^1^Department of Surgery, University of Alberta, 8440-112 Street, Edmonton, AB, Canada T6G 2B7; ^2^Center for the Advancement of Minimally Invasive Surgery (CAMIS), Royal Alexandra Hospital, Room 405 Community Services Center, 10240 Kingsway, Edmonton, AB, Canada T5H 3V9; ^3^Department of Surgery, Hopital Du Sacre Coeur, 191-315 Place D'Youville, Montreal, QC, Canada H2Y 0A4

## Abstract

The prevalence of morbidly obese individuals is rising rapidly. Being overweight predisposes patients to multiple serious medical comorbidities including type two diabetes (T2DM), hypertension, dyslipidemia, and obstructive sleep apnea. Lifestyle modifications including diet and exercise produce modest weight reduction and bariatric surgery is the only evidence-based intervention with sustainable results. Biliopancreatic diversion (BPD) produces the most significant weight loss with amelioration of many obesity-related comorbidities compared to other bariatric surgeries; however perioperative morbidity and mortality associated with this surgery are not insignificant; additionally long-term complications including undesirable gastrointestinal side effects and metabolic derangements cannot be ignored. The overall quality of evidence in the literature is low with a lack of randomized control trials, a preponderance of uncontrolled series, and small sample sizes in the studies available. Additionally, when assessing remission of comorbidities, definitions are unclear and variable. In this review we explore the pros and cons of BPD, a less well known and perhaps underutilized bariatric procedure.

## 1. Introduction

Global obesity, defined as a body mass index (BMI) greater than 30 kg/m^2^, is on the rise. Over the last thirty years mean BMI in individuals aging 20 years or older has increased at an escalating rate of 0.4 kg/m^2^ per decade [1]. This disease is a complex multisystem condition, associated with increased comorbidities including type two diabetes (T2DM), dyslipidemia, hypertension, obstructive sleep apnea, heart disease, stroke, asthma, bone and joint problems, cancer, and depression [2]. Cumulatively this has negative implications on health and longevity with the potential to reverse life-expectancy gains in high-income nations [3]. In fact, obesity is the fifth leading risk factor for mortality worldwide [4]. Lifestyle modifications, including diet and exercise, have been shown to be ineffective in treating obesity long term. Bariatric surgery has been shown to produce significant sustainable weight loss in obese patients. In addition, bariatric surgery has been effective not just for weight loss but also for improvement or remission of obesity-related comorbidities [5]. Of the various bariatric surgical procedure, biliopancreatic diversion (BPD) is considered the most effective in producing marked weight loss and comorbidity reduction [5]. However, concerns remain regarding the pros and cons with this surgical intervention in obese patients. This review will explore the literature supporting BPD and focus on the limitations of this technique.

## 2. Bariatric Surgery

The exponential rise in obesity has been matched by advances in surgical techniques of bariatric procedures [6, 7]. Traditionally, surgical procedures have been divided into restrictive, malabsorptive, or a combination of both. Restrictive procedures include vertical banded gastroplasty, laparoscopic adjustable gastric banding (LAGB), and laparoscopic sleeve gastrectomy (LSG). These techniques limit the size of the stomach thereby limiting caloric intake. Malabsorptive techniques divert biliopancreatic secretions, limiting the absorption of nutrients in the intestine. Jejunoileal bypass, popular in the 1960s and 1970s, is defined as a purely malabsorptive procedure. It has been abandoned due to significant morbidity and mortality [8, 9]. Biliopancreatic diversion, with (BPD/DS) or without (BPD) duodenal switch, and Roux-en-Y gastric bypass (RYGB) are defined as combination procedures, having both restrictive and malabsorptive features. Additionally, recent evidence suggests that gastrointestinal hormonal, inflammatory, central nervous system, and gut microbial factors contribute to overall benefits of these procedures [10–13].

## 3. Biliopancreatic Diversion

**Figure 1:**
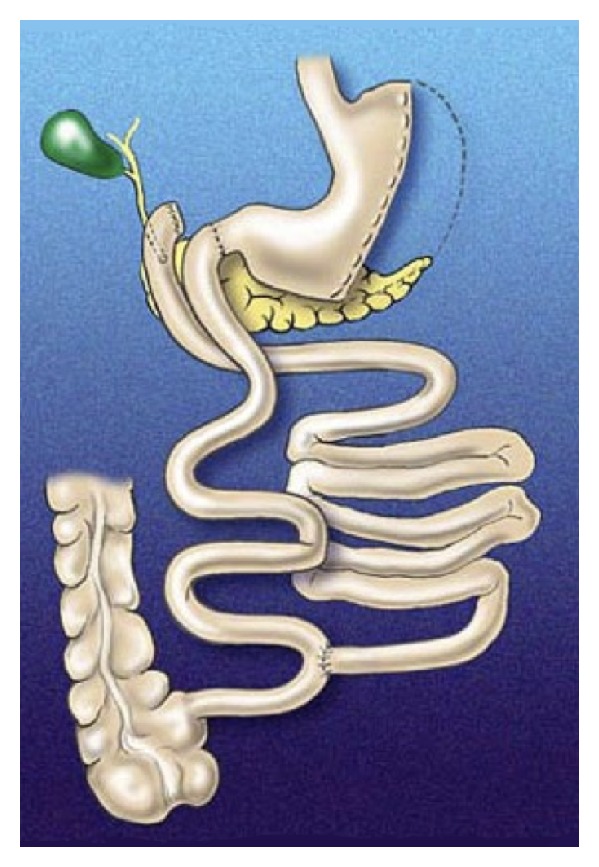
Illustration of the biliopancreatic diversion with duodenal switch procedure.

BPD was originally described by Scopinaro in 1979 as an alternative to jejunoileal bypass for severely obese patients [14]. The BPD procedure consists of: (1) partial distal gastrectomy in which the duodenal stump is closed (or bypass of the distal part of the stomach), (2) transection of the small bowel approximately halfway between the ligament of Treitz and the ileocecal valve, (3) Roux-en-Y gastroenterostomy from the gastric pouch to the distal bowel loop creating an alimentary limb, and (4) a biliopancreatic limb anastomosed with the alimentary limb 50 cm before the ileocecal valve forming a common channel [14]. The addition of the duodenal switch (DS), in which a vertical sleeve gastrectomy is combined with a duodenoenterostomy, was termed the “second generation BPD” [15]. DS involves preservation of the lesser curvature, antrum, pylorus, and first part of the duodenum along with lengthening of common channel lengths from 50 cm to 100 cm or more (Figure 1). These modifications were created to control for complications associated with Scopinaro's original description including marginal ulceration, vomiting, diarrhea, dumping syndrome, and micronutrient deficiencies. As such, Marceau et al. in a retrospective cohort study showed decreased necessity for revisional surgery secondary to aforementioned complications, specifically a 50% reduction in emesis and overall favourable micronutrient profiles [16]. Nonetheless, BPD/DS is one of the most complex and highest risk bariatric surgeries utilized today.

## 4. BPD/DS-Associated Weight Loss

BPD/DS has proven to be successful in achieving and maintaining significant weight loss in the superobese population (BMI >50 kg/m^2^). Buchwald's systematic review which included 48 studies (3 nonrandomized controlled trials, 6 comparative retrospective, 21 uncontrolled case series, 14 single-arm retrospective, and 3 observational studies) for a total of 1565 patients compared bariatric surgical procedures. This review suggests that BPD/DS is the most effective operation with a percentage of excess body weight loss (EBWL), defined as (preoperative BMI−current BMI)/(preoperative BMI−25) × 100, of 73% at 2 years follow-up, followed by gastric bypass (63%), gastroplasty (56%), and gastric banding (49%) [17]. Sovik et al. randomized 60 superobese patients (BMI 50–60 kg/m^2^) to undergo either RYGB or BPD/DS. Two years after surgery percentage of EBWL was found to be 31.2% following RYGB compared to 44.8% following BPD/DS. Additionally, health-related quality of life, as measured by the Short Form Health—36 survey, improved equally in both groups [18]. Similarly, Prachand et al. retrospectively analyzed 350 superobese patients who underwent either BPD/DS or RYGB. Preoperative BMI was significantly greater in the BPD/DS group compared to the RYGB group (58.8 kg/m^2^ versus 56.4 kg/m^2^, *P* = 0.0014). Percentage of EBWL was found to be significantly greater in the BPD/DS group compared to RYGB (12 months, 64.1% versus 55.9%; 18 months, 71.9% versus 62.8%; 24 months, 71.6% versus 60.1%; 36 months, 68.9% versus 54.9%) [19]. Contrarily, Deveney et al. compared weight loss after 1 and 2 years in super-obese patients who underwent RYGB or BPD/DS and reported percentage of EBWL to be similar between the 2 groups: 54% versus 53% at 1 year and 67% versus 64% at 2 years, with longer length of stay and higher rates of anastomotic leak in the BPD/DS group. It is important to note that those patients undergoing BPD/DS had higher BMI (59 kg/m^2^ versus 55 kg/m^2^, *P* < 0.05); therefore percentage of EBWL would be proportionally smaller when compared to smaller patients in the RYGB group. Percentage of EBWL being equal signifies greater overall weight loss in the BPD/DS group [20].

 Biertho et al. in an uncontrolled series of 810 morbidly obese patients with mean initial BMI of 44.2 ± 3.6 kg/m^2^ showed a EBWL of 76% maintained at 8.6 years follow-up concluding that BPD/DS was appropriate for non-super-obese patients [21]. Concordantly, Anthone et al. in an uncontrolled series including 701 BPD/DS patients with preoperative BMIs ranging from 34 kg/m^2^ to 95 kg/m^2^, found a EBWL of 69% after 1 year, 73% after 3 years, and 66% after 5 or more years of follow-up [22]. Overall, several uncontrolled series investigating BPD/DS suggest similar results with EBWL ranging from 61% to 85% with moderate-term follow-up [23–29]. 

## 5. BPD/DS and Cardiometabolic Risk Factors

BPD and BPD/DS have a marked effect on obesity-related comorbidities, specifically T2DM (Table 1) [17, 21, 24, 25, 30–38]. Mingrone et al. randomized 60 morbidly obese patients with T2DM to receive medical therapy (lifestyle modifications and hypoglycemic agents) or surgical therapy (RYGB or BPD). They reported no remission of T2DM in the medical therapy group, compared to 75% in the RYGB group and 95% in the BPD group after 2 years of follow-up [30]. Supportively, both Iaconelli et al.'s uncontrolled series including 50 patients and Tsoli et al.'s nonrandomized trial including 24 patients showed resolution of T2DM in all BPD patients 12 months after surgery [31, 32]. A recent systematic review and metaanalysis confirmed that diabetes resolution was the greatest for patients undergoing BPD/DS, followed by RYGB, and least for banding procedures [17]. Bariatric surgery has now been recommended for management of T2DM for select obese patients by the International Diabetes Federation [39]. Astiarraga et al. recently assessed the effect of BPD/DS on T2DM in nonobese patients demonstrating marked amelioration (improved glycemia) of metabolic control and remission (HbA_1*C*_ <6.5% and normal oral glucose tolerance test) in 1/3 of patients, suggesting a weight-independent effect of the operation, as only modest weight loss (−12 kg at 2 mo, −14 kg at 1 yr) was observed in this nonobese patient population [33]. 

Other cardiometabolic risk factors, including hypertension (Table 2) and dyslipidemia (Table 3), have also shown marked improvement following BPD/DS [21, 24, 25, 28, 29, 31, 32, 35, 36, 38, 40]. Additionally, obstructive sleep apnea was resolved in the majority of patients (Table 4) [21, 24, 29, 34, 38].

## 6. BPD/DS Related Complications

A recent paper by Buchwald and Oien, which surveyed International Federation for the Surgery of Obesity and Metabolic Disorders member nations with an 84% response rate, revealed that the proportion of BPD/DS procedures in relation to all bariatric surgeries declined from 6.1% to 4.9% to 2.1% in 2003, 2008, and 2011, respectively. Trends vary geographically. Overall, more BPD/DS procedures were executed in 2011 compared to 2008 and 2003; however other bariatric surgeries were performed preferentially [7]. This raises questions about why the procedure with the greatest-weight loss, evidence of lasting effect, and reversal of obesity-related comorbidities is the least performed bariatric surgery worldwide. The answer is likely multifactorial and complex. Firstly, the technical complexity of this procedure is a consideration, with the operation being time consuming and requiring a skilled surgeon. A laparoscopic approach, introduced by Gagner in 1999, sought the benefits of BPD/DS weight loss and reduced morbidity associated with laparoscopic surgery [41, 42]. In some studies this has proven to be true, with lower postoperative complication rates [43, 44]; however others show no difference when compared to an open procedure [45]. Likely, learning curve and operative volume may be important considerations, with a majority of BPD/DS being performed at focused speciality centers [46, 47]. 

### 6.1. Perioperative Mortality and Morbidity

In a recent meta-analysis of 361 studies including 85 048 patients overall mortality within 30 days of bariatric surgery was found to be 0.28%. BPD/DS had the highest early mortality with a rate of 0.29% to 1.23% for open and 0.0% to 2.7% for laparoscopic procedures [44]. Postoperative mortality is most commonly associated with pulmonary embolism, respiratory failure, and anastomotic leaks. It is important to acknowledge that BPD/DS is the procedure of choice for the most extremely obese patients; therefore it can be assumed that surgical risk in this group is higher at baseline. This was demonstrated in the Longitudinal Assessment of Bariatric Surgery Consortium, a prospective multicenter observational study, which included 4776 patients analyzing 30-day outcomes after bariatric surgery. It showed that extreme values of BMI were significantly associated with increased risk of major adverse outcomes (death; venous thromboembolism; percutaneous, endoscopic, or operative reintervention; and length of stay greater than 30 days) [48]. 

One-year complication rates as reported in the US Bariatric Outcomes Longitudinal Database are 4.6%, 10.8%, 14.9%, and 25.7%, respectively, after LAGB, LSG, RYGB, and BPD/DS [49]. This includes minor complications such as gastrointestinal side effects including flatulence, malodorous stools, and steatorrhea. Of major complications, gastrointestinal anastomotic leak is the most common, serious, early surgical complication. Hamoui et al. reviewed 701 BPD/DS cases performed over a ten-year period and reported that 5% of patients developed complications necessitating revisional surgery. Protein malnutrition was the most common indication for reoperation. A postoperative complication rate of 15% was then seen in their revisional surgery group, with wound infections being the most common complication in this group [50]. Biertho et al. analyzed a series of 1000 BPD/DS patients, in which major complications occurred in 7% of patients. They showed no difference in complication rate when comparing laparoscopic to open BPD/DS. Rehospitalization was required in 12.7% of patients and reoperations occurred in 6% of patients [43]. 

In a randomized trial of 60 patients, Sovik et al. compared mean operating time, median length of stay, and complication rates between RYGB and BPD/DS. On average RYGB required 91 min compared to 206 min for BPD/DS. Median length of stay was 2 days post-RYGB and 4 days post-BPD/DS. Perioperative complication rates were comparable between groups; however this study was likely underpowered and larger studies are necessary to truly draw conclusions [51]. It can not be understated that a greater volume of less complex bariatric procedures could be performed in the time that it takes to complete this operation. With the overwhelming burden of obesity and need for bariatric surgical procedures mindful resource allocation is crucial. A staged procedure with BPD/DS following LSG for select patients may allow for better utilization of resources in those patients who would benefit most from this complex surgery.

### 6.2. Metabolic Related Complications

BPD/DS is the bariatric procedure associated with some of the greatest perioperative malnutrition/metabolic related complications. All patients begin supplementation postoperatively; however there is no standardized approach to replacement and sometimes deficiencies are refractory to dietary supplements. Following BPD/DS patients can consume normal nutritional meals and continue to be malnourished [52]. Iron-deficiency anemia, protein calorie malnutrition, hypocalcemia, and deficiency of fat soluble vitamins, vitamin B1, vitamin B12, and folate are common [53]. BPD/DS has proven to be more malabsorptive compared to other bariatric surgeries; thus close follow-up is essential. As an example, the regimen implemented by Marceau et al. in their uncontrolled case series with fifteen-year follow-up was as follows: iron 300 mg, calcium 500 mg, vitamin D 50 000 IU, vitamin A 20 000 IU, 1 multivitamin tablet, and yogurt probiotics. Adjustments were made as appropriate; consequently severe anemia and vitamin deficiencies were uncommon [34]. Supplementation is of paramount importance; unfortunately in this patient population compliance is lacking [54].

Aasheim et al. randomized 60 super-obese patients to receive either RYGB or BPD/DS comparing 25-hydroxyvitamin D, vitamin A, and vitamin B1 up to 1 year postoperatively. BPD/DS patients had lower mean 25-hydroxyvitamin D and vitamin A concentrations, as well as a steeper decline in vitamin B1 compared to RYGB [55]. Decreased vitamin D and calcium levels with associated secondary hyperparathyroidism have been demonstrated [56–58]. Marceau et al., after prospectively analyzing 33 patients utilizing iliac crest bone biopsy, bone mineral density, and biochemical investigations, proclaimed that despite serum abnormalities in vitamin D, calcium, and PTH overall bone mineral density and fracture risk were unchanged 10 years after BPD/DS [54]. A population-based retrospective cohort study out of the United Kingdom confirmed these results, concluding that bariatric surgery (60% gastric band, 29% RYGB, 11% other—BPD/DS was not separated) did not significantly effect fracture risk with a mean follow-up of 2.2 years. Additionally, facture risk was independent of the specific surgical technique. Longer-term studies are necessary to ensure that results are enduring [59]. Clinically there have been case reports of BPD/DS-related vitamin A deficiency and associated night-blindness [60, 61], post-BPD/DS peripheral neuropathies associated with B12 deficiencies [61], and Wernicke's encephalopathy as a result of B1 deficiencies [62, 63]. 

## 7. Conclusion

Current evidence suggests that BPD/DS produces the greatest weight loss in obese individuals with the most significant improvement in obesity-related comorbidities. However, the utilization of this bariatric surgical procedure is limited compared to other surgical options. The technical complexity of BPD/DS and lack of knowledge may only partly explain the decreased utilization by surgeons. Concern regarding severe metabolic disturbances and malnutrition may also be implicated. Currently, BPD/DS remains a reasonable surgical option for severely obese patients, especially those with a BMI > 50 kg/m^2^ when performed by expert surgeons in high volume speciality centers with close follow-up. Additionally, BPD/DS should be considered as a staged procedure following LSG for select patients who would benefit most from this complex surgery. There continues to be a need to educate surgeons regarding this viable bariatric surgical procedure to foster accurate knowledge regarding BPD/DS as the potentially positive metabolic effects outweigh the known risks as supported by evidence available in the body of literature.

## Figures and Tables

**Table 1:** Effect of biliopancreatic diversion with duodenal switch on diabetes.

Study *n* (number of patients)	DM incidence preoperatively	Resolution of DM	Measure of resolution
Buchwald et al. [17] 31 studies of BPD *n* = 2502	30%	95%	Clinical and laboratory manifestations of diabetes—not otherwise specified (different measures for individual studies)

Biertho et al. [21] *n* = 810	28%	93%	Discontinuation of diabetic treatment (oral hypoglycemic agents/insulin)

Crea et al. [24] *n* = 540			Discontinuation of diabetic treatment (oral hypoglycemic agents/insulin) within 1 yr
A-BPD: 287	A-35%	A-98%	
B-DS: 253	B-36%	B-64%	

Papadia et al. [25] *n* = 68	3%	100%	Fasting glucose < 125 mg/dL

Mingrone et al. [30] *n* = 60			Discontinuation of diabetic treatment (oral hypoglycemic agents/insulin), fasting glucose < 100 mg/dL, and HbA1C < 6.5%
A-medical: 20	A-100%	A-0%	
B-RYGB: 20	B-100%	B-75%	
C-BPD: 20	C-100%	C-95%	

Iaconelli et al. [31] *n* = 50			Not defined
A-medical: 28	A-100%	A-45%	
B-BPD: 22	B-100%	B-100%	

Tsoli et al. [32] *n* = 24			Oral glucose tolerance test
A-BPD: 12	A-100%	A-100%	
B-SG: 12	B-100%	B-100%	

Astiarraga et al. [33] *n* = 30	50%	40%	HbA1C < 6.5% and normal oral glucose tolerance test

Marceau et al. [34] *n* = 1423	28%	92%	Discontinuation of oral hypoglycemic agents Discontinuation of insulin
		98%	

Vage et al. [35] *n* = 80	100%	94%	Fasting glucose < 7 mmol/L and HbA1C < 6.5%

Dorman et al. [36] *n* = 329			Self-reported
A-BPD/DS: 173	A-36%	A-82%	
B-RYGB: 139	B-31%	B-64%	

Prachand et al. [37] *n* = 350			Discontinuation of the medications used for treatment with the absence of symptoms
A-BPD/DS: 198	A-24%	100%	
B-RYGB: 152	B-36%	60%	

Pata et al. [38] *n* = 874	35%	97%	Discontinuation of oral hypoglycemic agents Discontinuation of insulin
		67%	

DM: diabetes mellitus.

BPD: biliopancreatic diversion.

DS: duodenal switch.

RYGB: Roux-en-Y gastric bypass.

SG: sleeve gastrectomy.

**Table 2:** Effect of biliopancreatic diversion with duodenal switch on hypertension.

Study *n* (number of patients)	Hypertension incidence preoperatively	Resolution of hypertension	Measure of resolution
Biertho et al. [21] *n* = 810	37%	60%	Discontinuation of antihypertensive medications

Crea et al. [24] *n* = 540			Not defined
A-BPD: 287	A-55%	A-93%	
B-DS: 253	B-52%	B-94%	

Papadia et al. [25] *n* = 68	49%	82%	Discontinuation of antihypertensive medications and blood pressure < 140/85 mmHg

Baltasar et al. [29] *n* = 125	8%	90%	Not defined

Iaconelli et al. [31] *n* = 50			Blood pressure < 130/85 mmHg
A-medical: 28	A-71%	A-25%	
B-BPD: 22	B-64%	B-73%	

Vage et al. [35] *n* = 80	84%	54%	Discontinuation of antihypertensive medications and blood pressure < 140/90 mmHg

Dorman et al. [36] *n* = 329			Self-reported
A-BPD/DS: 190	A-58%	A-67%	
B-RYGB: 139	B-57%	B-39%	

Prachand et al. [37] *n* = 350			Discontinuation of the medications used for treatment with the absence of symptoms
A-BPD/DS: 198	A-67%	A-68%	
B-RYGB: 152	B-37%	B-39%	

Pata et al. [38] *n* = 874	57%	95%	Discontinuation of antihypertensive medications and blood pressure < 140/90 mmHg

BPD: biliopancreatic diversion.

DS: duodenal switch.

RYGB: Roux-en-Y gastric bypass.

**Table 3:** Effect of biliopancreatic diversion with duodenal switch on dyslipidemia.

Study *n* (number of patients)	Dislipidemia incidence preoperatively	Resolution of dyslipidemia	Measure of resolution
Crea et al. [24]	A-Hypercholesterolemia 87%	A-Hypercholesterolemia 98%	Hypercholesterolemia/hypertriglyceridemia—Laboratory values within the normal range (not defined)
*n* = 540	A-Hypertriglyceridemia 53%	A-Hypertriglyceridemia 97%	
A-BPD: 287	B-Hypercholesterolemia 85%	B-Hypercholesterolemia 99%	
B-DS: 253	B-Hypertriglyceridemia 55%	B-Hypertriglyceridemia 99%	

Papadia et al. [25] *n* = 68	16%	100%	Serum cholesterol < 200 mg/dL

Vage et al. [35] *n* = 80	48% (on treatment)	92%	LDL Hyperlipidemia—discontinuation of hypolipidemic agents with LDL < 2.6 mmol/L

Dorman et al. [36] *n* = 329			Hyperlipidemia—not defined
A-BPD/DS: 190	A-54%	A-81%	
B-RYGB 139	B-44%	B-55%	

Prachand et al. [37] *n* = 350			Dyslipidemia—discontinuation of the medications used for treatment with the absence of symptoms
A-BPD/DS: 198	A-31%	A-72%	
B-RYGB: 152	B-36%	B-26%	

Pata et al. [38] *n* = 874	Hypercholesterolemia 87% Hypertriglyceridemia 53%	Hypercholesterolemia 98% Hypertriglyceridemia 96%	Laboratory values within the normal range (cholesterol 120–200 mg/dL, triglycerides < 150 mg/dL)

BPD: biliopancreatic diversion.

DS: duodenal switch.

Medical: medical management of weight loss/comorbidities.

Tcholesterol: total cholesterol.

TG: triglycerides.

HDL: high-density lipoprotein.

LDL: low-density lipoprotein.

RYGB: Roux-en-Y gastric bypass.

**Table 4:** Effect of biliopancreatic diversion with duodenal switch on obstructive sleep apnea.

Study *n* (number of patients)	Obstructive sleep apnea incidence preoperatively	Resolution of obstructive sleep apnea	Measure of resolution
Biertho et al. [21] *n* = 810	25%	98%	Discontinuation of breathing apparatus

Crea et al. [24] *n* = 540			Discontinuation of CPAP apparatus
A-BPD: 287	A-7%	A-100%	
B-DS: 253	B-6%	B-100%	

Baltasar et al. [29] *n* = 125	6%	100%	Not defined

Marceau et al. [34] *n* = 1423	40%	90%	Discontinuation of CPAP apparatus

Pata et al. [38] *n* = 874	9%	100%	Discontinuation of breathing apparatus

BPD: biliopancreatic diversion.

DS: duodenal switch.

CPAP: continuous positive airway pressure.
